# Adenocarcinoma of the Rete Testis: A Case Report

**DOI:** 10.7759/cureus.22210

**Published:** 2022-02-14

**Authors:** Charles F Mitchell, Shane Pearce

**Affiliations:** 1 Urology, Elson S. Floyd College of Medicine, Washington State University, Spokane, USA; 2 Urology, Spokane Urology, Spokane, USA

**Keywords:** urologic pathology, urologic oncology, urology, rete testis adenocarcinoma, rete testis, adenocarcinoma

## Abstract

Adenocarcinoma of the rete testis is an extremely rare and aggressive tumor that carries a poor prognosis. Successful long-term treatment for such tumors remains elusive as more cases are discovered worldwide. Treatment typically involves radical orchiectomy, retroperitoneal pelvic lymph node dissection, adjuvant chemotherapy, and/or continued surveillance. Here we describe the case of a 42-year-old male with a history of low testosterone who presented with a localized adenocarcinoma of the left rete testis. He was treated with radical orchiectomy and continued surveillance alone due to a lack of evidence of metastasis on follow-up imaging. History, prognosis, diagnostics, and treatment guidelines, as well as the most significant recent cases since the last rete testis adenocarcinoma literature meta-analysis, are discussed.

## Introduction

Adenocarcinoma of the rete testis is a rare, aggressive testicular tumor with a mortality rate of 46% and a median survival time of 33 months [1]. Less than 80 cases have been reported in the English literature since it was first described by Feek and Hunter in 1945 [1-3]. These tumors are most commonly seen in middle-aged white men with a mean age of 53 years, although ages have varied from 17 to 91 years. Symptomatic presentation is non-specific, with approximately 36% of known cases before 2019 having an associated hydrocele and 46% having a palpable mass/swelling, but only 16% endorsing pain. Pathological architecture also varies greatly, with papillary, tubulopapillary, and glandular most commonly described; moreover, 69% of known cases show mixed growth patterns [1]. Diagnostic imaging has proven inconsistent in detecting metastases, and nearly all treatments outside of radical orchiectomy have shown mixed utility in overall survival [1,4]. Given the rarity of the tumor, varied architectural and symptomatic presentation, and poor diagnostic imaging efficacy, it is predominantly a diagnosis of exclusion. Here, we present a case of a low-grade adenocarcinoma of the rete testis without any evidence of metastatic disease on initial staging or postoperative imaging.

## Case presentation

A 42-year-old white male with a history of low testosterone was referred by his primary care physician for a progressively enlarging left testicular mass. The patient reported first noticing the mass two years prior. He had no history of hydrocele, varicocele, testicular pain, flank pain, or prior scrotal surgery. Physical examination revealed a non-tender palpable left testicular mass without scrotal swelling.

**Figure 1:**
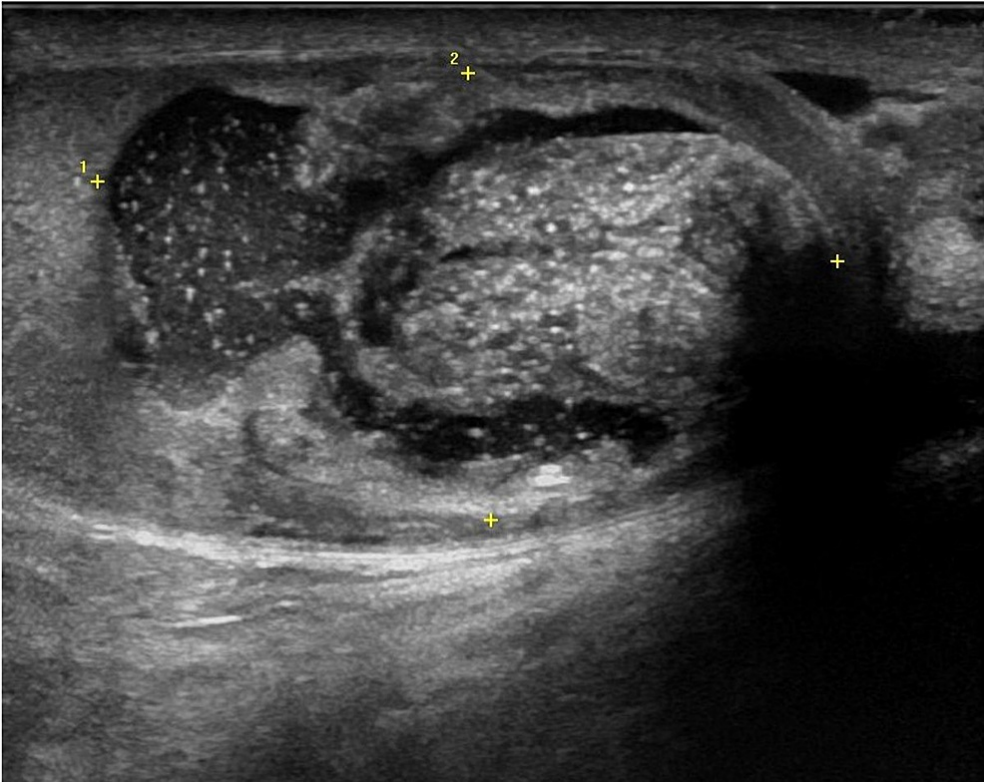
Ultrasound of the testes showing a left testicular mass (encompassed within the yellow plus symbols).

**Figure 2:**
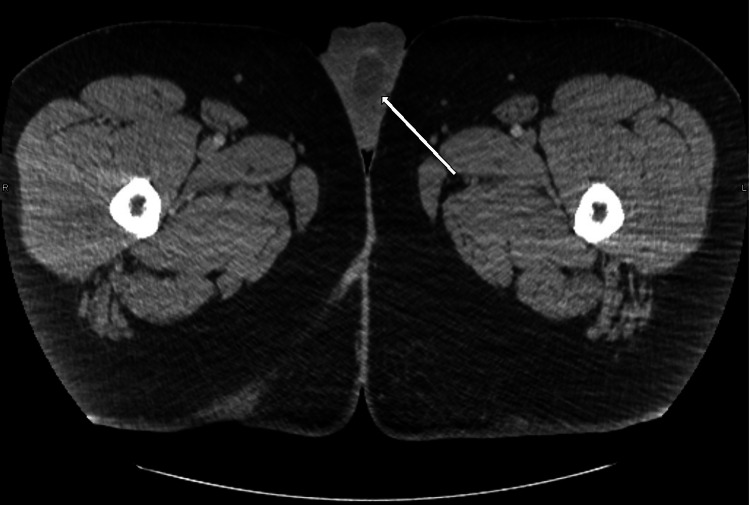
CT of the scrotum showing a left testicular mass (white arrow). CT: computerized tomography

A scrotal ultrasound revealed a heterogeneous 3.8 × 2.3 × 3 cm mass present in the left testicle which demonstrated internal Doppler flow and no abnormal scrotal thickening (Figure 1). A subsequent abdominal and pelvic computerized tomography (CT) scan demonstrated a left testicular mass with no pelvic or retroperitoneal lymphadenopathy or other sites of metastasis (Figure 2). Relevant lab values obtained at this time included total testosterone of 143 ng/dL, a percentage free testosterone of 6.10%, total prostate-specific antigen (PSA) of 0.2 ng/mL, and free PSA of 25.0%. After careful counseling regarding the risks and benefits of surgery, the patient underwent an uneventful left radical orchiectomy.

Pathology revealed a low-grade adenocarcinoma of the rete testis measuring 3.8 cm in its largest dimension, which was confirmed upon review by a genitourinary pathologist at a high-volume academic institution. Immunohistochemical staining revealed diffusely positive staining for paired box gene 8 (PAX8), hepatocyte nuclear factor-1 beta (HNF1-β), cytokeratin (CK) 7, thyroid transcription factor 1 (TTF-1), and CD10. The tissue stained negative for steroidogenic factor 1 (SF-1), CK5 and 6, Wilms tumor 1 (WT1), BRCA1-associated protein 1 (BAP1), GATA binding protein 3 (GATA3), and α-inhibin. Ki-67 index staining revealed a low proliferation rate (Table 1). The tumor was determined to be malignant with low-grade cytologic features emanating from the rete testis. Differential diagnosis included left testis adenoma, rete testis adenocarcinoma, clear cell adenocarcinoma of the testis, and metastatic carcinoma; however, pathology favored rete testis origin and low-grade adenocarcinoma based on location, immunoprofile, and morphology.

**Table 1:** Immunohistochemical staining and postoperative follow-up serum tumor markers.

	Result
Immunohistochemical stain	
GATA binding protein 3 (GATA3)	-
α-Inhibin	-
Steroidogenic factor 1 (SF-1)	-
Wilms tumor 1 (WT1)	-
Cytokeratin 5 (CK5)	-
Cytokeratin 6 (CK6)	-
Cytokeratin 7 (CK7)	+
BRCA1-associated protein 1 (BAP1)	-
Paired-box gene 8 (PAX8)	+
Common acute lymphoblastic leukemia antigen (CD10)	+
Hepatocyte nuclear factor-1 beta (HNF1-β)	+
Thyroid transcription factor 1 (TTF1)	+
Antigen KI-67 (Ki-67)	Low
Serum tumor marker	
Serum testosterone	205 ng/dL
Alpha-fetoprotein (AFP)	1.3 ng/mL
Lactate dehydrogenase (LDH)	176 IU/L
Human chorionic gonadotropin (HCG)	<1 mIU/mL
Free prostate-specific antigen (PSA)	0.2 ng/mL

The case was presented at a multidisciplinary tumor board after orchiectomy and outside of pathology review. The consensus recommendation was for active surveillance as opposed to primary retroperitoneal pelvic lymph node dissection (RPLND), adjuvant radiation, or chemotherapy. The patient consented to this plan and was educated on the importance of continued surveillance. A four-month postoperative CT scan demonstrated a thickened scrotal wall with an empty left post-orchiectomy space and no evidence of metastatic disease. His tumor markers, including lactate dehydrogenase, alpha-fetoprotein, human chorionic gonadotropin, and PSA, remained negative (Table 1). Total testosterone remained low at 205 ng/dL during this time. The patient was living well at postoperative month 30 but was lost to follow-up.

## Discussion

Nochomovitz and Orenstein established the exclusion criteria for adenocarcinoma of the rete testis in 1984 [5]. Their requirements were as follows: absence of histologically similar extrascrotal tumor that could be the primary site; tumor centered in the hilum of the testis; morphology incompatible with any other type of testicular or paratesticular tumor; and immunohistochemical exclusion of other possibilities. Even among strict criteria, erroneous diagnosis is common, requiring strict immunohistochemical guidelines to rule out mesotheliomas, serous adenocarcinomas of the ovarian type, and other germ cell tumors.

Our case met the above Nochomovitz and Orenstein criteria. Immunohistochemical stains for antigens often positive in primary testicular germ cell and sex cord-stromal tumors (GATA3, α-inhibin, SF-1) were negative in the neoplastic cells. Malignant mesothelioma was excluded due to the specimen’s negative staining for WT1, CK5/6, and BAP1, and positive staining for CK7, PAX8, CD10, and a low Ki-67 index. Cell morphology and subsequent pre/postoperative imaging scans excluded ovarian-type clear cell carcinoma and metastatic adenocarcinoma of the lung despite positive staining for HNF1-β and TTF1. Our specimen’s immunohistochemical profile was consistent with a recent study by Al-Obaidy et al. [1], who performed a systematic review of the English literature before 2019 alongside their six-patient case series.

Ten new cases of rete testis adenocarcinoma have been reported in the English language literature since Al-Obaidy et al. in 2019, seven of which were discovered by the Al-Obaidy team [6]. Three of such cases have offered novel diagnostic and surveillance techniques. Arnesen et al. [7] identified an 85-year-old patient with a history of elevated PSA but negative prostate biopsy. Their tumor specimen notably stained positive for NK3 homeobox 1 (NKX3.1), which was not yet positive in any previously reported cases, and P501S. Their sample had a morphology reminiscent of grade 4 prostatic adenocarcinoma or intraductal prostatic adenocarcinoma, but this diagnosis was ruled out via immunohistochemical staining. Their study adds NKX3.1 to the list of stains to consider and highlights the risk of mistaking adenocarcinoma of the rete testis for metastatic prostate adenocarcinoma.

Similarly, Kitano et al. [8] identified carbohydrate antigen 19-9 (CA 19-9) as a potential new biomarker for monitoring rete testis adenocarcinoma metastasis. Their case revealed a 44-year-old-man whose serum CA 19-9 levels rose as their tumor metastasis progressed. Pani et al. [9] recently reported a case believed to be the longest known follow-up in the English literature, which found that annual 2-deoxy-2 (F18) fluoro-D-glucose (FDG) positron emission tomography combined with CT (FDG PET/CT) can identify metastasis for yearly resections. While annual imaging and surgical resection may not be feasible for all patients, their case highlights the need for constant surveillance and aggressive treatment following radical orchiectomy.

Despite these new diagnostic and surveillance techniques, optimal treatment for adenocarcinoma of the rete testis remains unclear due to its rarity and late presentation. A meta-analysis of 40 cases performed by Sanchez-Chapado et al. found a greater three-year survival rate in those who received RPLND than those who did not (83% vs. 42%, log-rank P = 0.03) [10]. As noted by the authors, however, RPLND was predominantly performed on patients without evidence of visceral metastasis, introducing potential selection bias. Despite these findings, modern systematic reviews have not found statistically significant benefits for greater survival between RPLND and orchiectomy alone (P = 0.2) [1]. Tumor size has been reported as a prognostic factor [11]; however, reanalysis of the original data lacked statistical significance [1]. Evidence has demonstrated that all chemotherapy regimens attempted to date have shown no statistical benefit to overall survival [1,4,8,11-13]. Tumor confinement to the testis appears to be the only proven predictor of survival, with an 18% three-year survival in patients with metastasis. Having an obvious transition from normal to malignant rete testis epithelium is likewise a beneficial diagnostic directive.

Given the uncertainty of optimal treatment protocol, our patient followed conservative management post-radical orchiectomy. His rare histologic subtype indicated annual PSA surveillance to rule out metastatic prostate cancer and a CT of the lung to rule out primary lung cancer, both of which remained unremarkable for 30 months until he was lost to follow-up. Adjuvant radiation has traditionally not been recommended, as in this case, but it could be considered in advanced disease [4,12]. The absence or presence of metastasis is the only consistent predictor of overall survival (P < 0.001 [11] and P = 0.02 [1]). With this in mind, primary RPLND was determined to be of minimal benefit given the patient’s normal imaging, tumor markers, and low-grade primary tumor.

## Conclusions

Adenocarcinoma of the rete testis is a rare tumor that requires fastidious pathological examination and immunohistochemical staining for accurate diagnosis. The absence or presence of metastasis appears to be the only consistent predictor of survival. Given the variability in presentation and treatment approaches, it is difficult for a clinician to determine the best diagnostic workup for a suspected adenocarcinoma of the rete testis. Standard testicular tumor workup which includes immunohistochemical staining for mesothelioma and adenocarcinomas of the lung, prostate, and ovarian types should be conducted. Outside review of pathology should be encouraged, as well as a multidisciplinary management approach with input from urology, radiation oncology, and medical oncology. The development of guidelines regarding a standard immunohistochemical regiment for suspected tumors and a follow-up treatment protocol are recommended to help standardize evaluation and future care.
